# Heterologous Expression in Remodeled *C*. *elegans*: A Platform for Monoaminergic Agonist Identification and Anthelmintic Screening

**DOI:** 10.1371/journal.ppat.1004794

**Published:** 2015-04-30

**Authors:** Wenjing Law, Leah M. Wuescher, Amanda Ortega, Vera M. Hapiak, Patricia R. Komuniecki, Richard Komuniecki

**Affiliations:** Department of Biological Sciences, The University of Toledo, Toledo, Ohio, United States of America; University of Georgia, UNITED STATES

## Abstract

Monoamines, such as 5-HT and tyramine (TA), paralyze both free-living and parasitic nematodes when applied exogenously and serotonergic agonists have been used to clear *Haemonchus contortus* infections *in vivo*. Since nematode cell lines are not available and animal screening options are limited, we have developed a screening platform to identify monoamine receptor agonists. Key receptors were expressed heterologously in chimeric, genetically-engineered *Caenorhabditis elegans*, at sites likely to yield robust phenotypes upon agonist stimulation. This approach potentially preserves the unique pharmacologies of the receptors, while including nematode-specific accessory proteins and the nematode cuticle. Importantly, the sensitivity of monoamine-dependent paralysis could be increased dramatically by hypotonic incubation or the use of *bus* mutants with increased cuticular permeabilities. We have demonstrated that the monoamine-dependent inhibition of key interneurons, cholinergic motor neurons or body wall muscle inhibited locomotion and caused paralysis. Specifically, 5-HT paralyzed *C*. *elegans* 5-HT receptor null animals expressing either nematode, insect or human orthologues of a key Gα_o_-coupled 5-HT_1_-like receptor in the cholinergic motor neurons. Importantly, 8-OH-DPAT and PAPP, 5-HT receptor agonists, differentially paralyzed the transgenic animals, with 8-OH-DPAT paralyzing mutant animals expressing the human receptor at concentrations well below those affecting its *C*. *elegans* or insect orthologues. Similarly, 5-HT and TA paralyzed *C*. *elegans* 5-HT or TA receptor null animals, respectively, expressing either *C*. *elegans* or *H*. *contortus* 5-HT or TA-gated Cl^-^ channels in either *C*. *elegans* cholinergic motor neurons or body wall muscles. Together, these data suggest that this heterologous, ectopic expression screening approach will be useful for the identification of agonists for key monoamine receptors from parasites and could have broad application for the identification of ligands for a host of potential anthelmintic targets.

## Author Summary

Monoamines, such as serotonin (5-HT) and tyramine (TA), paralyze both free-living and parasitic nematodes when applied exogenously. Since nematode cell lines are not available and animal screening options are limited, we have developed a screening platform to identify monoamine receptor agonists that involves the heterologous expression of key receptors from parasitic nematodes in chimeric, genetically-engineered mutant *C*. *elegans*, at sites likely to yield robust phenotypes upon agonist stimulation. Specifically, we have demonstrated that agonist dependent activation of Gα_o_-coupled 5-HT receptors or monoamine-gated Cl^-^ channels in key interneurons, cholinergic motor neurons or body wall muscle inhibited locomotion and caused paralysis. This approach includes nematode-specific accessory proteins and the nematode cuticle, and appears to preserve the unique pharmacologies of the individual receptors. Together these data highlight the utility of these transgenic *C*. *elegans* for agonist identification and their potential for anthelmintic screening.

## Introduction

Nematode infections cause significant morbidity and contribute significantly to a loss of Disability Adjusted Life Years (DALYs) [1–4]. For example, soil-transmitted nematodes, including *Necator americanus*, *Trichuris trichuris* and *Ascaris lumbricoides* infect nearly 2 billion worldwide and are a source of disease in over 400 million children [5]. More importantly, in many cases, such as filarial infection, effective chemotherapy is still not available [6]. Parasitic nematodes also have a devastating economic impact in agricultural settings that, at least secondarily, contributes significantly to a decline in human welfare, especially in areas where good nutrition is already compromised. For example, parasitic nematodes infect livestock and major crops (corn and soybeans) and cause billions in economic losses yearly in the US alone [7]. Importantly, most commercially available anthelmintics have become increasingly ineffective because of growing resistance (benzimidazoles, levamisole and, most recently, ivermectin) and most nematicides (DCBP (1,2-dibromo-3-chloropropane), methyl bromide), to control plant nematodes, have been banned by the EPA because of human toxicity [8–13]. New drugs, new drug targets and new, more effective screening protocols are desperately needed in all settings.

Most anthelmintics in use today act as **agonists** at key receptors and cause paralysis by interfering with muscle contraction and/or locomotion [14–17]. Since receptor “activation” is essential for anthelmintic activity, receptor knockout is not necessarily the “gold standard” for target validation; in fact knockout may not be lethal. Five molecular targets have been used for drug discovery, two nicotinic cholinergic receptor subunits (tetrahydropyrimidines/imidathiazoles and amino-acetonitriles), glutamate-/GABA-gated Cl^-^ channels (macrocyclic lactones and piperazine, respectively) and Ca^++^-gated K^+^ channels (emodepside) [14–17]. Importantly, each of these anthelmintics is active in the free-living nematode, *Caenorhabditis elegans* and our understanding of their modes of action has, in large part, resulted from our ability to genetically manipulate their putative targets in receptive *C*. *elegans* mutant backgrounds [18–21]. Importantly, the identification of new targets has been limited by the lack of useful information about the identity, function and localization of the additional receptors regulating muscle contraction and locomotion. In addition to identifying new targets, we also need new screening protocols that preserve the unique pharmacologies of the receptors from the different parasites and maintain a nematode-specific context that includes the cuticle and appropriate accessory proteins, especially given that no nematode cells lines are available and that the parasites themselves are extremely difficult and expensive to culture.

In the present study, we have developed a heterologous, ectopic over-expression approach to provide a unique nematode screening platform for selective agonist identification, exploiting the unique experimental advantages of the *C*. *elegans* model system. Previously, we and others have demonstrated that exogenous monoamines, such as serotonin (5-HT), dopamine (DA) and tyramine (TA), each paralyze *C*. *elegans* and, where examined, parasitic nematodes [22–33]. In each case, the key *C*. *elegans* receptors mediating this locomotory inhibition have been identified and functionally localized, with each operating at a different level within the locomotory circuit: 5-HT in a few key interneurons, including the two AIB interneurons, DA in the cholinergic motor neurons and TA in head muscle and additional interneurons associated with locomotory decision-making [24, 28, 30]. We have previously constructed quintuple 5-HT receptor null *C*. *elegans* (5-HT *quint*) that do not express any previously identified 5-HT receptors and do not respond to exogenous 5-HT, to identify essential roles for the Gα_o_-coupled 5-HT_1_-like SER-4 and the unique 5-HT-gated Cl^-^ channel, MOD-1 in 5-HT-dependent locomotory paralysis [23, 24]. Importantly, SER-4 agonists appear to function as anthelmintics *in vivo* and have been used to clear *Haemonchus contortus* infections from gerbils [34, 35]. In the present study, we ectopically expressed SER-4 and MOD-1 orthologues from parasitic nematodes, insects and humans in either the cholinergic motor neurons or body wall muscles of quintuple *C*. *elegans* 5-HT receptor null animals that lack all known *C*. *elegans* 5-HT receptors, on the assumption that agonist-dependent receptor activation at these sites will cause robust phenotypes that can be readily adapted for agonist screening. For example, the activation of a ligand-gated Cl^-^ channel in body wall muscles would be predicted to hyperpolarize the muscle and significantly inhibit locomotion, while the activation of a Cl^-^ channel or Gα_o_-coupled GPCR on the cholinergic motor neurons would significantly inhibit acetylcholine (ACh) release from the motor neurons and inhibit both muscle contraction and thus, locomotion. Importantly, as noted below, both hypotheses have proven to be correct.

## Materials and Methods

### Strains and reagents

*bus-8* (*e2968*), *bus-16* (*e2802*) and *bus-17* (*e2800*) were obtained from *Caenorhabditis* Genetics Center (CGC). *ser-5* (*tm2654*)*;ser-4* (*ok512*)*;mod-1* (*ok103*)*;ser-7* (*tm1325*) *ser-1* (*ok345*) (5-HT *quint*), *ser-5* (*tm2654*)*;mod-1* (*ok103*)*;ser-7* (*tm1325*) *ser-1* (*ok345*) (SER-4 *quad*) and *lgc-55* (*tm2913*);*tyra-3* (*ok325*) *tyra-2* (*tm1846*) *ser-2* (*pk1357*) (TA *quad*) were generated as described previously [23, 24]. All strains were maintained on NGM plates with *OP50* at 16°C. The cDNA clone of *Drosophila melanogaster* 5-HT1A (RE57708) was ordered from the *Drosophila* Genomics Resource Center (DGRC), the cDNA clone Human HTR1A (MGC: 167873; clone ID: 9020250) from GE Healthcare Dharmacon Inc. and cDNA clones of *Haemonchus contortus* (Hco) *lgc-55* and *mod-1* orthologues were kindly provided by Dr. Sean Forrester [33, 36]. The *unc-17ß* promoter, RM#621p, was obtained from Dr. James Rand. The integrated AIB::HisCl1 in N2 (*cx15457*) animals were a kind gift from Dr. Cornelia Bargmann [37].

Serotonin (5-HT) (H7752-25G), tyramine (TA) (T2879-25G), 8-OH DPAT (H141-25MG), sumatriptan succinate (S1198-10MG), PAPP (S009-25MG) and histamine dihydrochloride (H7250-5G) were purchased from Sigma Life Sciences. Stock solutions (50 mM) of 5-HT, TA, 8-OH-DPAT, sumatriptan and histamine were made up in distilled water, PAPP in 100% ethanol. The constituent of for nematode growth media (NGM), potassium phosphate monobasic (KH_2_PO_4_; P285-3), sodium chloride (NaCl; S271-3), calcium chloride dehydrate (CaCl_2_.2H_2_O; C79-500), magnesium sulfate heptahydrate (MgSO_4_.7H_2_O; BP213-1), tryptone (BP1421-2) and agar (DF0812071) were purchased from Thermo Fisher Scientific Inc., cholesterol (C3045-5G) purchased from Sigma Life Science.

### Fusion PCR and transgenic lines

All transgenic constructs were created by overlap fusion PCR [38]. All transgenes contain a GFP marker (with *unc-54* 3′-UTR) at the 3’-end. Primers used are listed in S1 Text. PCR products from multiple reactions were pooled and co-injected with coelomocyte-RFP screening marker into the appropriate null backgrounds [39]. Once generated, transgenic animals are frozen in liquid nitrogen and thawed fresh weekly for assay. Multiple transgenic lines from each construct were examined.

### Paralysis assay

Fresh agar plates (without NaCl, KH_2_PO_4_, MgSO_4_, CaCl_2_, tryptone and cholesterol) containing 5-HT, TA, PAPP, sumatriptan or 8-OH DPAT at desired concentrations were made daily. For assays involving *bus* mutants, fresh NGM agar plates (with NaCl, KH_2_PO_4_, MgSO_4_, CaCl_2_, tryptone and cholesterol) containing 5-HT were used for all assays. For assays with AIB::HisCl1 (*cx15457*) animals, freshly poured NGM agar or agar only plates containing 10 mM and 2 mM histamine were used. NGM agar plates were prepared as described in WormBook [40].

For all paralysis assays, well-fed, transgenic young adults expressing RFP screening markers were picked 2 hrs prior to assay and maintained on NGM plates with *E*. *coli OP50*. For assay, 10 animals are transferred to assay plates (agar only for all assays and NGM agar for assays with *bus* mutants) containing the appropriate drug and motility was assessed at intervals of 5 min for 30 min. Experiments with sumatriptan were carried out for 60 min, with motility assessed every 5 min. All assays were conducted in the absence of food, *i*.*e*. *OP50*. Animals that moved less than 1 body bend/20 s were counted as paralyzed. Each transgenic line was assayed at least 3 times with 10 animals/assay for each agonist concentration. Data is presented as % paralyzed ± SE over drug exposure time (min). Dose-response curves and EC_50_s were then generated using a variable slope nonlinear regression model with GraphPad Prism 6 software. Drug concentrations were log10-transformed prior to analysis.

### Accession numbers

The accession numbers of the proteins involved in our study are *C*. *elegans* SER-4 (accession no. NP_497452), *C*. *elegans* LGC-55 (accession no. NP_507870), *C*. *elegans* MOD-1 (accession no. CCD72364), *D*. *melanogaster* 5-HT1A (accession no. NM_166322.2), *D*. *melanogaster* HisCl1 (accession no. Q9VGI0), human HTR1A (accession no. BC136263), *H*. *contortus* LGC-55 (accession no. ACZ57924.1) and *H*. *contortus* MOD-1 (accession no. ADM53350.1).

## Results

### Rationale

The monoamines, 5-HT, DA and TA each dramatically inhibit locomotion in *C*. *elegans* when applied exogenously at concentrations high enough to overcome the permeability barrier of the nematode cuticle, ultimately resulting in paralysis [24, 25, 27, 30]. Using the *C*. *elegans* model, the receptors involved in monoamine-dependent locomotory inhibition have been identified and localized [22–30]. Interestingly, the key receptors involved in 5-HT, DA and TA inhibition each function at a different level in the locomotory circuit with 5-HT-dependent paralysis requiring the expression of the Gα_o_-coupled, 5-HT_1_-like receptor, SER-4, and the 5-HT-gated Cl^-^ channel, MOD-1 in a limited number of interneurons, including the two AIBs [24, 25]. Unfortunately, since nematode cell lines are not available and the maintenance of parasitic nematodes outside their hosts is problematic, screening platforms for anti-nematodal activity have been limited and do not usually incorporate the nematode cuticle or potentially important nematode accessory proteins.

The present study was designed to develop a screening platform for nematode monoamine receptor agonists in “chimeric” genetically-engineered *C*. *elegans* by heterologously expressing 5-HT and TA receptors at sites likely to yield robust phenotypes upon agonist stimulation. Previously, many investigators have rescued a range of behaviors in *C*. *elegans* null animals with the expression of proteins from the parasites, validating this approach [41–43]. We chose to examine locomotion as an endpoint for heterologous, ectopic expression, as the neurons and circuits modulating locomotion in *C*. *elegans* and parasitic nematodes appear to be conserved, can be readily assessed by established screening assays, and have always been the primary target for the majority of existing anthelmintics. Specifically, we expressed 1) Gα_o_-coupled, 5-HT_1_-like receptors, or 5-HT/ TA-gated Cl^-^ channels in the cholinergic motor neurons of *C*. *elegans* mutants lacking any 5-HT or TA receptors, respectively on the assumption that robust agonist-dependent Gα_o_ signaling or potential hyperpolarization, respectively, would dramatically inhibit ACh release and locomotion and 2) 5-HT or TA-gated Cl^-^ channels in body muscle of *C*. *elegans* mutants lacking any 5-HT or TA receptors, respectively, on the assumption that agonist-dependent muscle hyperpolarization would cause paralysis.

### 5-HT inhibits locomotion in 5-HT receptor null animals expressing 5-HT_1_-like receptors in the AIB interneurons or cholinergic motor neurons

The role of the *C*. *elegans* 5-HT_1_-like receptor, SER-4, in 5-HT-dependent paralysis is well documented [23–25, 44]. Indeed, the utility of the *H*. *contortus* SER-4 orthologue, 5-HT_1HC_ as an anthelmintic target has been validated previously both *in vivo* and *in vitro* [34, 35]. Locomotion in *C*. *elegans* has been assessed previously using a number of different assays, many of which can be readily adapted for screening [45–50]. For example, automated thrashing assays allow thousands of compounds to be easily screened per day [48]. Monoamine-dependent locomotory inhibition and paralysis has been quantified on agar plates (sinusoidal body bends) and in liquid medium (C-shaped “swimming”), containing either M9 buffer or water [22, 24, 25, 27, 29, 30]. The permeability of the *C*. *elegans* cuticle appears to vary depending on incubation conditions, with much less 5-HT apparently required in water, than in salt-containing media (M9), possibly because of an increased cuticular permeability under hypotonic conditions [25].

**Fig 1:**
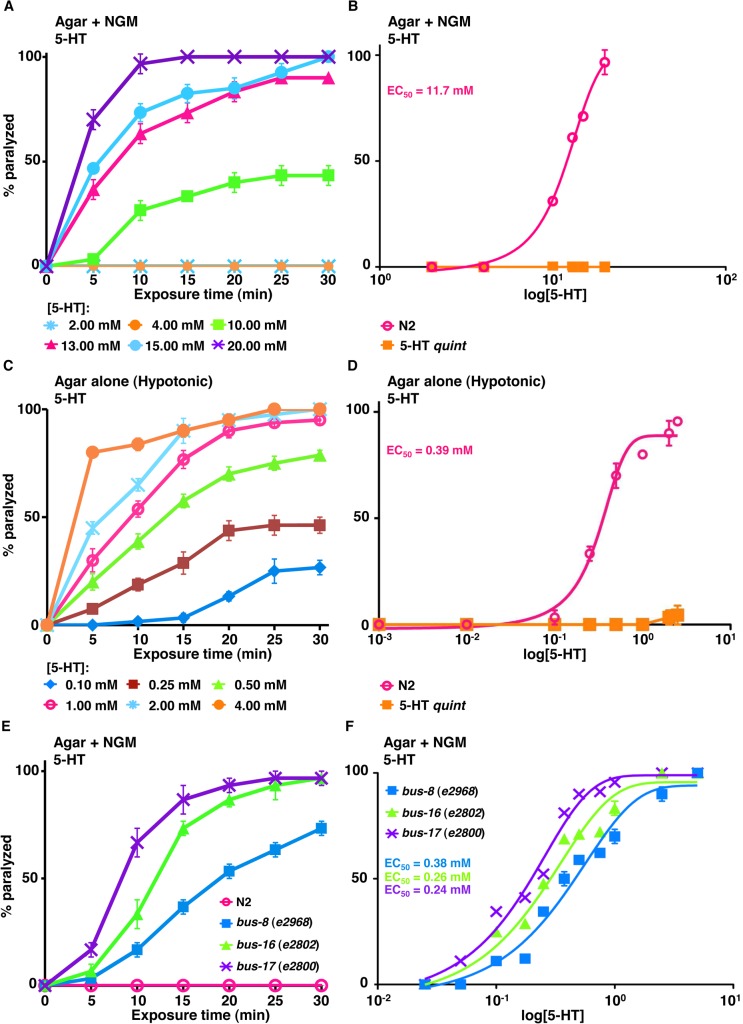
*C*. *elegans* mutants with increased cuticular permeability are hypersensitive to 5-HT-dependent paralysis. **A-B.** Paralysis of wild type and mutant *C*. *elegans* on NGM agar plates. **A.** Wild type animals examined for 5-HT-dependent paralysis as outlined in Methods. Data are presented as mean ± SE (n = 3). **B.** Dose-response curves for 5-HT-dependent paralysis on NGM plates at 10 min exposure for wild type and 5-HT *quint* animals. **C-D.** Paralysis of wild type and mutant *C*. *elegans* on non-NGM agar (hypotonic) plates. **C.** Wild type animals were examined for 5-HT-dependent paralysis as outlined in Methods. Data are presented as mean ± SE (n = 3). **D.** Dose-response curves for 5-HT-dependent paralysis in hypotonic conditions at 15 min exposure for wild type and 5-HT *quint* animals. **E-F.** 5-HT-dependent paralysis of wild type and mutant *C*. *elegans* on NGM agar plates. **E.** 5-HT (0.25 mM)-dependent paralysis of wild-type, *bus-8* (*e2968*), *bus-16* (*e2802*) and *bus-17* (*e2800*) mutants. Data are presented as mean ± SE (n = 3). **F.** Dose-response curves for 5-HT-dependent paralysis at 10 min exposure for wild type and *bus* mutants.

Previously, we assayed locomotion under standard *C*. *elegans* culture conditions on NGM agar plates. Under these conditions, 15 mM 5-HT initiated a rapid paralysis in wild type animals, and *ser-5;mod-1;ser-7 ser-1* quadruple null (SER-4 *quad*) animals [24, 44]. As predicted, 5-HT had no effect on locomotion in 5-HT *quint* animals that lack all previously identified 5-HT receptors (Fig 1A and 1B) [24]. This 5-HT-dependent paralysis was not the classical spastic paralysis associated with cholinergic agonists, such as levamisole, or the flaccid paralysis associated with glutamatergic agonists, such as ivermectin, but instead appeared to result more from “locomotory confusion,” with animals unable to effectively integrate conflicting sensory inputs to initiate and sustain forward/backward locomotion. The *C*. *elegans* cuticle appears to be more impermeable than those of some of the parasitic nematodes [51–53]. Therefore, since the concentration of 5-HT required for maximal paralysis was quite high (15 mM) in these short term assays, presumably to overcome cuticular permeability, we re-assayed these animals under hypotonic conditions on agar plates without salt (non-NGM) (Fig 1C and 1D). Attempts to repeat published data from others on 5-HT paralysis in water were unsuccessful, as majority of the animals burst soon (within 5 min) after exposure to water [25]. However, in a hypotonic environment (agar alone without NGM), much lower concentrations of 5-HT were required for inhibition of wild type animals, with 1 mM 5-HT yielding 50% paralysis after 10 min exposure (EC_50_ about 0.4 mM) (Fig 1C and 1D).

In addition to hypotonic incubation, we also examined 5-HT-dependent paralysis in a number of *C*. *elegans* mutants that exhibit increased cuticular permeability. For example, the Hodgkin group previously identified a series of *bus* mutants that exhibit increased cuticular permeability that have been hypothesized to be excellent vehicles for small molecule screening [54]. Indeed, as noted in Fig 1E and 1F, many of the *bus* mutants are hypersensitive to 5-HT-dependent paralysis, even under isotonic assay conditions (on NGM agar plates). For example, *bus-17* mutants are acutely paralyzed after 10 min on 5-HT with an EC_50_ of about 0.24 mM, which is substantially lower than that observed in wild-type animals incubated under the same conditions (EC_50_ = 11.5 mM) (Fig 1F). These results suggest that these mutants might be useful for agonist identification, especially when only limited amounts of compound are available. Indeed, it may even be possible to select mutants that exhibit cuticular permeabilities that mimic those of individual parasites. Unfortunately, these mutants are also sensitive to hypotonicity and burst under the hypotonic conditions used in the present study, so that they could not be used in combination with hypotonicity to further increase sensitivity. Therefore, unless specified, hypotonic conditions were used to assay the transgenic animals described below.

**Fig 2:**
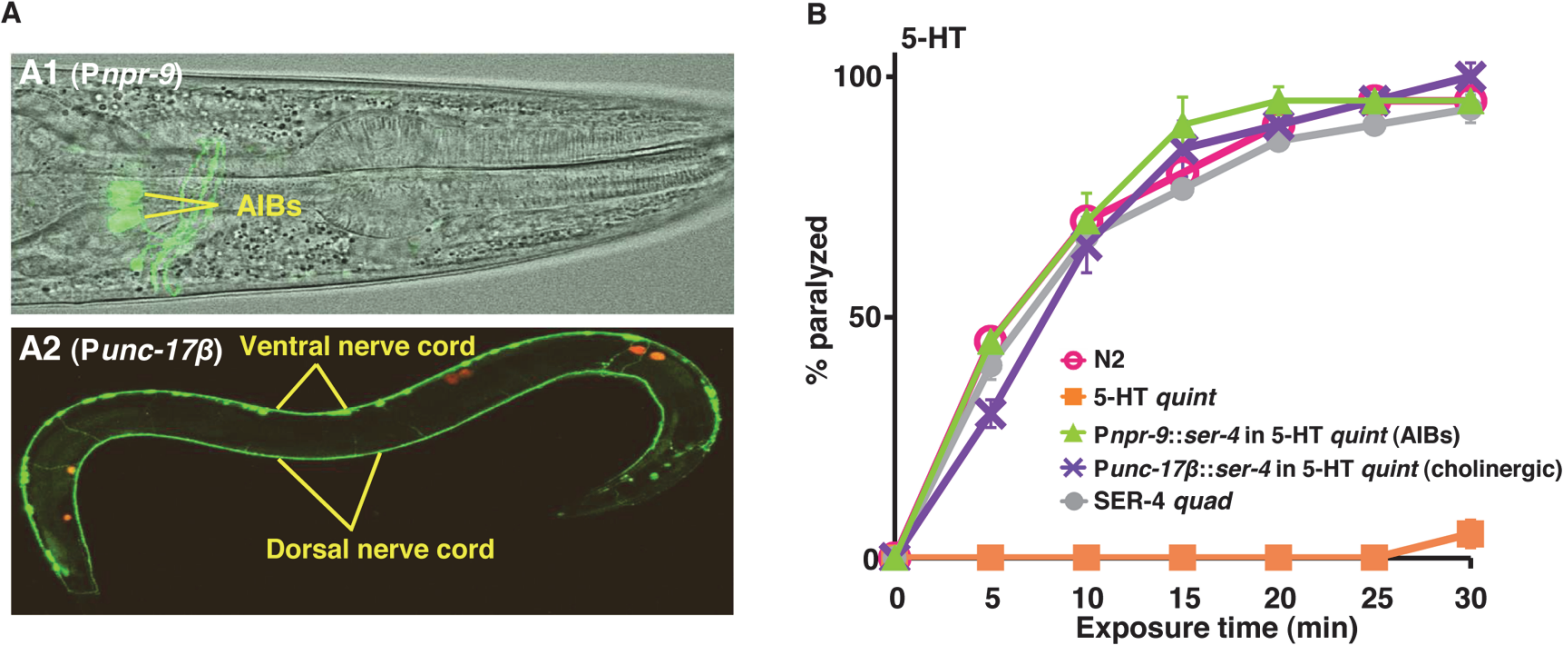
The 5-HT/SER-4-dependent inhibition of either the AIB interneurons or cholinergic motor neurons causes locomotory paralysis. **A.** Confocal images of 5-HT *quint* expressing SER-4::GFP in the AIB interneurons (P*npr-9*)(A1) or cholinergic motor neurons (P*unc-17β*)(A2). GFP fluorescence (A2) or GFP fluorescence overlaid on DIC image (A1). The red stain in A2 is coelomocyte-specific RFP screening marker. **B.** Paralysis of wild type, mutant and transgenic *C*. *elegans* on hypotonic, non-NGM agar plates. Wild type, quadruple 5-HT receptor null animals expressing only SER-4 (SER-4 *quad*) or 5-HT *quint* expressing the *C*. *elegans* 5-HT_1_-like receptor, SER-4, in either the cholinergic motor neurons (P*unc-17β*) or the two AIB interneurons (P*npr-9*) were examined for 5-HT (1 mM)-dependent paralysis as outlined in Methods. Data are presented as mean ± SE (n = 3).

A *ser-4*::*gfp* transgene is expressed in a limited number of neurons, including the AIBs [25]. Therefore, SER-4::GFP was specifically expressed in either the AIB interneurons (P*npr-9*) or ectopically, in the cholinergic motor neurons (P*unc-17β*) of the 5-HT *quint*. Expression was confirmed by GFP fluorescence (Fig 2A). As predicted, 5-HT *quint* animals expressing SER-4 in either the AIBs or cholinergic motor neurons were rapidly paralyzed by 5-HT (Fig 2B). Interestingly, on 5-HT, although 5-HT *quint* animals expressing SER-4 in the AIBs alone moved only infrequently, they initiated backward locomotion for a short distance when prodded with a blunt platinum wire at the tail, suggesting that they were probably unable to process conflicting locomotory signals, as hypothesized above. In contrast, animals expressing SER-4 in the cholinergic motor neurons were fully paralyzed and did not move when prodded.

### Use of heterologous expression for agonist identification

**Fig 3:**
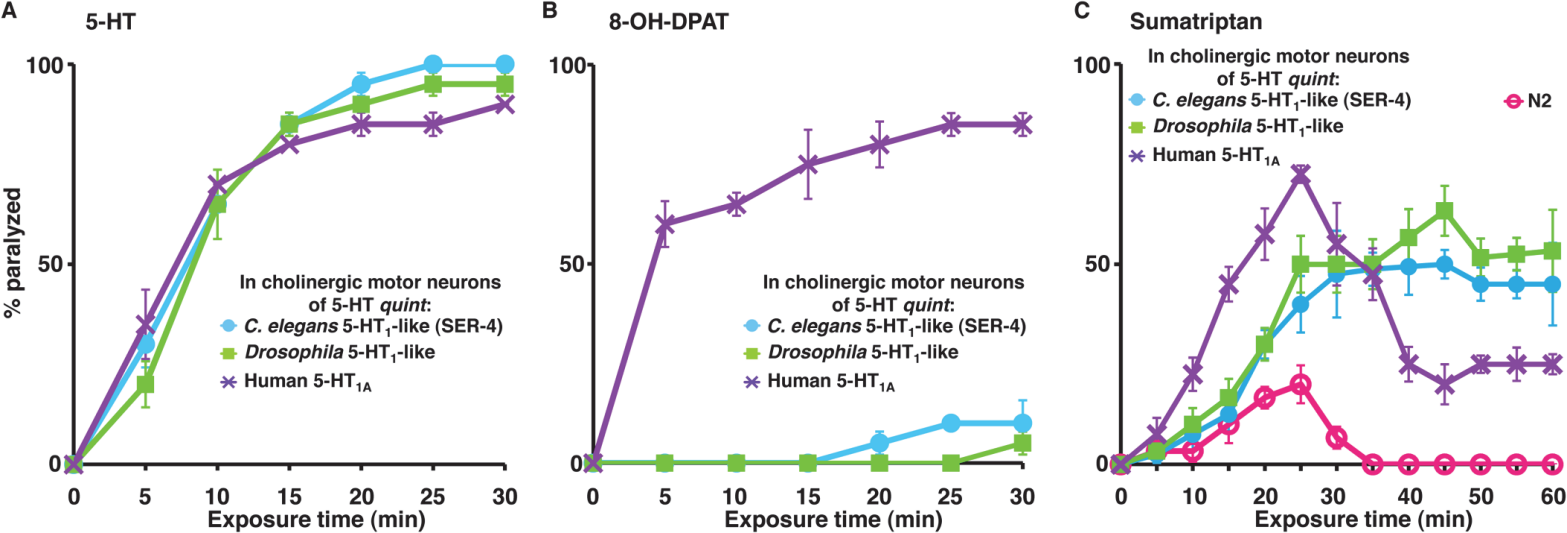
5-HT and 5-HT receptor agonists selectively paralyze *C*. *elegans* 5-HT receptor mutant animals expressing nematode, insect or human 5-HT_1_-like receptors in the cholinergic motor neurons. **A-C.** Paralysis of wild type, mutant and transgenic *C*. *elegans* on hypotonic, non-NGM agar plates. **A.** 5-HT (1 mM)-dependent paralysis of 5-HT *quint* animals expressing either *C*. *elegans* 5-HT_1_-like (SER-4), *Drosophila* 5-HT_1_-like, or human 5-HT_1A_ receptor in cholinergic motor neurons (P*unc-17β*). Data are presented as mean ± SE (n = 3). **B.** 8-OH-DPAT (2 mM)-dependent paralysis of 5-HT *quint* animals expressing either *C*. *elegans* 5-HT_1_-like (SER-4), *Drosophila* 5-HT_1_-like, or human 5-HT_1A_ receptor in cholinergic motor neurons (P*unc-17β*). Data are presented as mean ± SE (n = 3). **C.** Sumatriptan (1 mM)-dependent paralysis of wild type, 5-HT *quint* animals expressing either *C*. *elegans* 5-HT_1_-like (SER-4), *Drosophila* 5-HT_1_-like, or human 5-HT_1A_ receptor in cholinergic motor neurons (P*unc-17β*). Data are presented as mean ± SE (n = 3).

To demonstrate the utility of this screening approach, the *Drosophila* 5-HT_1_ orthologue (5HT1A) or the human 5-HT-1_A_ receptor (HTR1A) were also expressed specifically in the cholinergic motor neurons (P*unc-17β*) of 5-HT *quint* animals. Locomotion in animals from both transgenic lines was dramatically inhibited by exogenous 5-HT, demonstrating that the receptors were functionally expressed (Fig 3A). To demonstrate the specificity of these chimeric *C*. *elegans* for agonist identification, we examined the effect of 8-hydroxy-2-(di-n-propylamino)tetralin (8-OH-DPAT), a subtype-selective agonist for the human 5-HT_1A_ receptor, sumatriptan succinate, a selective mammalian 5-HT_1B/D_ agonist, and p-amino-phenethyl-m-trifluoromethylphenyl piperazine (PAPP). As predicted, 8-OH-DPAT rapidly paralyzed the 5-HT *quint* animals expressing the human 5-HT_1A_ receptor (Fig 3B). In contrast, 8-OH-DPAT, even at 2 mM, had no effect on locomotion 5-HT *quint* animals expressing either *Drosophila* or *C*. *elegans* 5-HT_1_ receptor orthologues, suggesting the conservation of ligand-receptor specificity in chimeric *C*. *elegans* (Fig 3B). Sumatriptan, at low concentrations, is a selective mammalian 5-HT_1B/D_ agonist, and, indeed in the present study, sumatriptan was much less effective than 8-OH-DPAT in initiating paralysis [55]. For example, 0.5 mM sumatriptan had no effect on locomotion in either wild type or transgenic animals expressing 5-HT_1A_ receptor orthologues in cholinergic motor neurons and, even at higher concentrations, failed to fully paralyze animals expressing the human 5-HT_1A_ receptor. In addition, although animals expressing the human 5-HT_1A_ receptor responded to increased sumatriptan concentrations more rapidly, these locomotory effects were transient and reduced dramatically after 25 min, presumably due to receptor desensitization (Fig 3C). In contrast, paralysis increased with prolonged sumatriptan exposure in animals expressing either the *C*. *elegans* or *Drosophila* receptors, demonstrating kinetic differences between the orthologous receptors.

**Fig 4:**
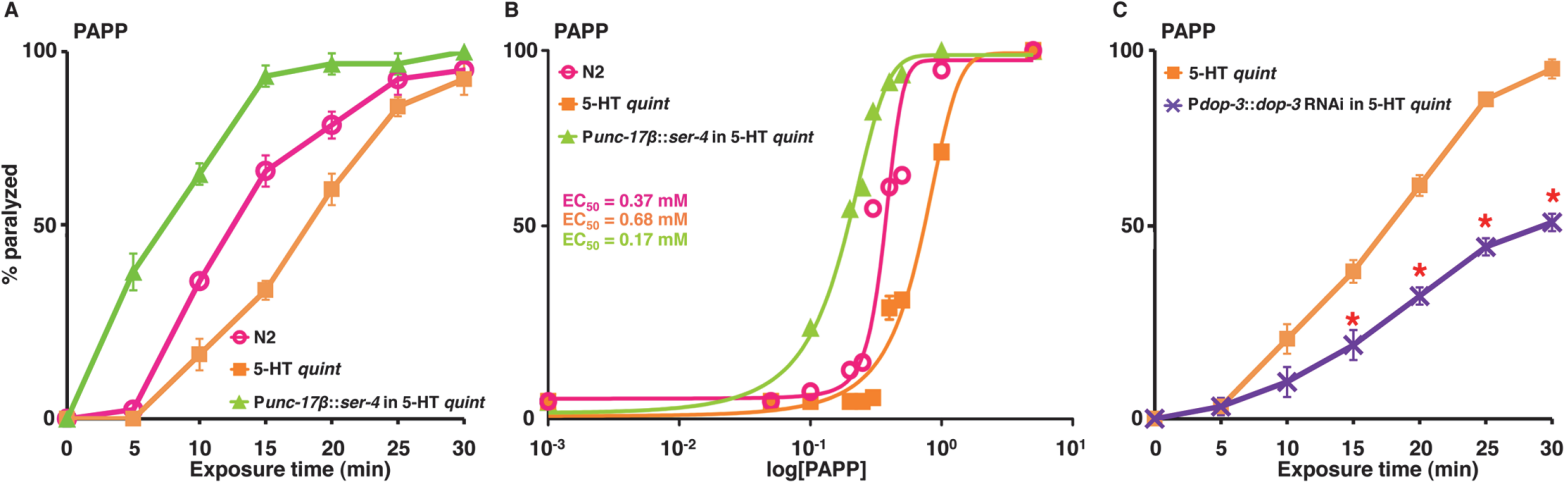
PAPP paralyzes *C*. *elegans* via SER-4 and DOP-3. **A-C.** Paralysis of wild type, mutant and transgenic *C*. *elegans* on hypotonic non-NGM agar plates. **A.** PAPP (0.5 mM)-dependent paralysis of wild-type, 5-HT *quint* and 5-HT *quint* animals expressing SER-4 in the cholinergic motor neurons (P*unc-17β*). Data are presented as mean ± SE (n = 3). **B.** Dose-response curves for PAPP-dependent paralysis at 15 min exposure for wild type, 5-HT *quint* and 5-HT *quint* animals expressing SER-4 in the cholinergic motor neurons (P*unc-17β*). **C.** PAPP (0.5 mM)-dependent paralysis of 5-HT *quint* and 5-HT *quint* animals expressing P*dop-3*::*dop-3* RNAi. Data are presented as mean ± SE (n = 3). ‘*’ p≤0.001, significantly different from 5-HT *quint* animals assayed under identical conditions.

PAPP, a high affinity agonist for the *H*. *contortus* 5-HT_1_-like receptor, paralyzes *H*. *contortus* L3s *in vitro* and clears experimental *H*. *contortus* infections from gerbils [34, 35]. As predicted, PAPP initiated a rapid paralysis in wild type animals (EC_50_ = 0.37 mM) and, even more rapidly, in 5-HT *quint* animals expressing the *C*. *elegans* SER-4 in the cholinergic motor neurons (EC_50_ = 0.17 mM), supporting the previous identification of PAPP as a 5-HT_1_-like receptor agonist (Fig 4A and 4B). In contrast, and somewhat surprisingly, at higher concentrations (≥0.5 mM), PAPP also paralyzed 5-HT *quint* animals (EC_50_ = 0.68 mM) that were unaffected by 5-HT, suggesting that, in addition to acting as a 5-HT_1_-like receptor (SER-4) agonist, PAPP also acted at second target(s) (Fig 4A and 4B). Since exogenous TA and DA also paralyze *C*. *elegans*, we surmised that, at higher concentrations, PAPP might be activating additional monoamine receptors. DA-dependent paralysis requires the expression of the Gα_o_-coupled DA receptor, DOP-3 in the cholinergic motor neurons [26]. Therefore, *dop-3* expression was knocked down in the 5-HT *quint* animals using *dop-3* RNAi driven by the *dop-3* promoter. As noted in Fig 4C, *dop-3* RNAi knockdown in this background significantly reduced PAPP-dependent paralysis, suggesting that DOP-3 is a secondary PAPP target. Screening is in progress to identify additional target(s). Together, these data highlight the utility of this approach in preliminary drug screening and suggest that it may also be useful for the identification of nematode-specific agonists.

### The activation of monoamine-gated Cl^-^ channels in cholinergic motor neurons or body wall muscles causes locomotory paralysis

**Fig 5:**
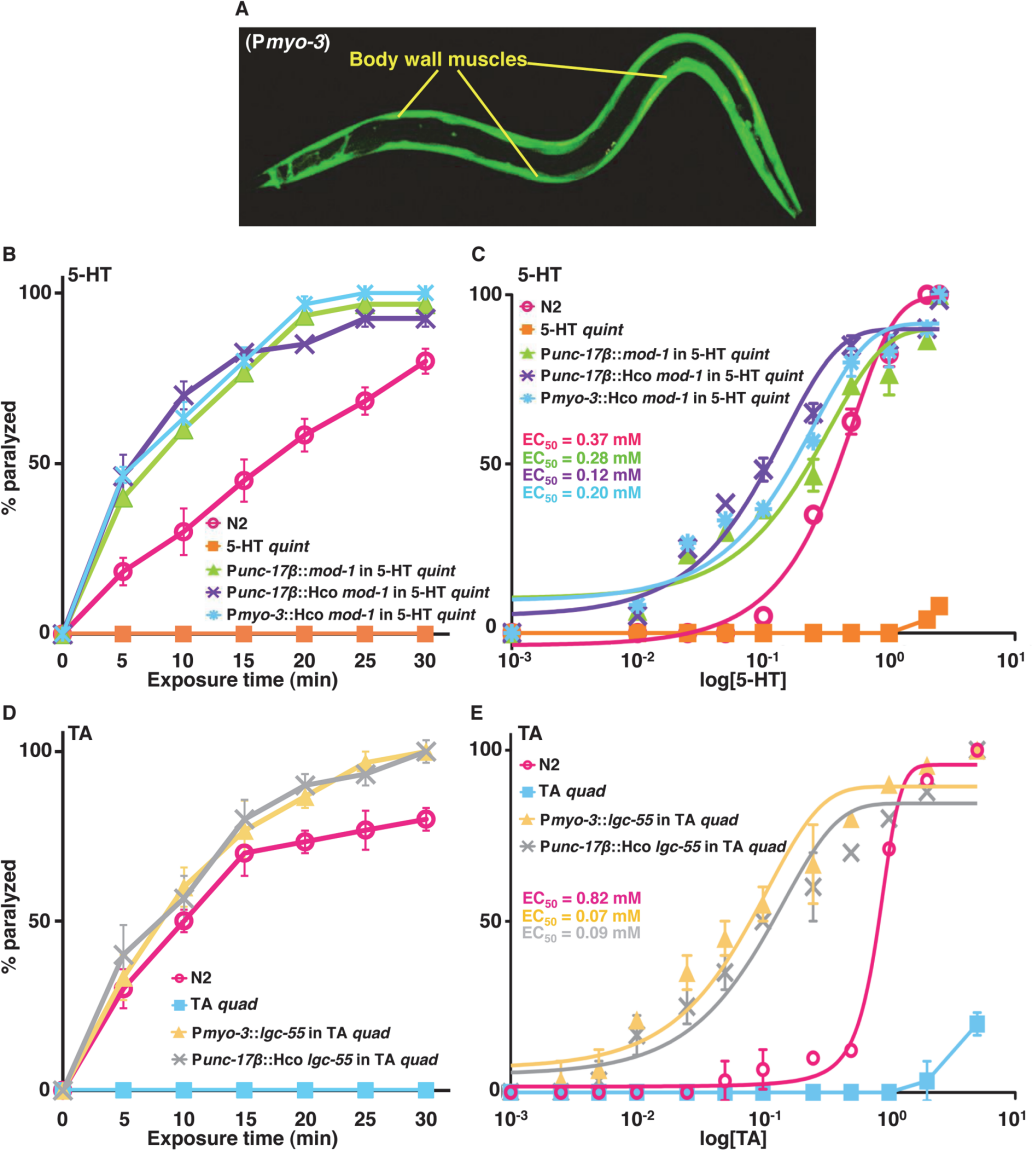
Exogenous monoamines paralyze *C*. *elegans* expressing monoamine-gated Cl^-^ channels in either cholinergic motor neurons or body wall muscles. **A.** Confocal image of 5-HT *quint* animals expressing *H*. *contortus* (Hco) MOD-1::GFP in body wall muscles (P*myo-3*). GFP-fluorescence image. **B-E.** Paralysis of wild type, mutant and transgenic *C*. *elegans* on non-NGM agar plates. **B.** 5-HT (0.5 mM)-dependent paralysis of wild type, 5-HT *quint* and 5-HT *quint* animals expressing either the *C*. *elegans* or *H*. *contortus* (Hco) MOD-1 orthologues in the cholinergic motor neurons (P*unc-17β*) or the *H*. *contortus* (Hco) MOD-1 orthologue in body wall muscle (P*myo-3*). Data are presented as mean ± SE (n = 4). **C.** Dose-response curves for 5-HT-dependent paralysis at 15 min exposure for wild type, 5-HT *quint* and 5-HT *quint* animals expressing either the *C*. *elegans* or *H*. *contortus* (Hco) MOD-1 orthologues in the cholinergic motor neurons (P*unc-17β*) or the *H*. *contortus* (Hco) MOD-1 orthologue in body wall muscle (P*myo-3*). **D.** Tyramine (1 mM)-dependent paralysis of wild type, TA *quad* and TA *quad* animals expressing either the *C*. *elegans* LGG-55 in body wall muscle (P*myo-3*) or the *H*. *contortus* (Hco) LGC-55 orthologue in the cholinergic motor neurons (P*unc-17β*). Data are presented as mean ± SE (n = 3). **E.** Dose-response curves for TA-dependent paralysis at 15 min exposure for wild type, TA *quad* and TA *quad* animals expressing either LGC-55 in the body wall muscles (P*myo-3*), or *H*. *contortus* (Hco) LGC-55 orthologue in cholinergic motor neurons (P*unc-17β*).

Nematodes also express a unique family of monoamine-gated Cl^-^ channels that appear to be highly conserved within the phylum, including the *C*. *elegans* 5-HT- and TA-gated Cl^-^ channels, MOD-1 and LGC-55, that play key roles in 5-HT- and TA-dependent muscle paralysis, respectively. The *C*. *elegans* MOD-1 and its *H*. *contortus* orthologue were expressed directly in either cholinergic motor neurons (P*unc-17β*) or body wall muscles (P*myo-3*) of 5-HT *quint* animals and 5-HT-dependent paralysis was assayed as described above. Muscle expression was confirmed by GFP fluorescence (Fig 5A). As previously noted, 5-HT had no effect on locomotion in 5-HT *quint* animals, but rapidly paralyzed the 5-HT *quint* animals expressing either the *C*. *elegans* MOD-1 in the cholinergic motor neurons or the *H*. *contortus* (Hco) MOD-1 orthologue in cholinergic motor neurons or body wall muscle, with EC_50_s of about 0.3 mM, 0.2 mM and 0.2 mM, respectively (Fig 5B and 5C). Interestingly, 5-HT-dependent paralysis was more rapid in the transgenic animals expressing MOD-1 orthologues in the cholinergic motor neurons than in wild type animals.

Similarly, LGC-55 was expressed in the body wall muscles (P*myo-3*) or its *H*. *contortus* orthologue in the cholinergic motor neurons (P*unc-17β*) of *lgc-55*;*tyra-3 tyra-2 ser-2* quadruple TA receptor null (TA *quad*) animals. TA *quad* animals lack all previously identified TA receptors and fail to respond to TA in a range of behavioral assays, including locomotion. As predicted, TA had no effect on locomotion in the TA *quad* animals, but significantly inhibited locomotion in TA *quad* animals expressing either *C*. *elegans* LGC-55 in body wall muscles or *H*. *contortus* (Hco) LGC-55 orthologue in cholinergic motor neurons, each with EC_50_ of about 0.1 mM (Fig 5D and 5E). Together, these data suggest that monoaminergic activation of these Cl^-^ channels hyperpolarizes either the cholinergic motor neurons or body wall muscles and inhibits muscle contraction, as well as highlighting the utility of chimeric *C*. *elegans* as a functional expression platform to identify ligand-gated Cl^-^ channels agonists for use as anthelmintics.

### The inhibition of AIB signaling causes “locomotory confusion” and paralysis

**Fig 6:**
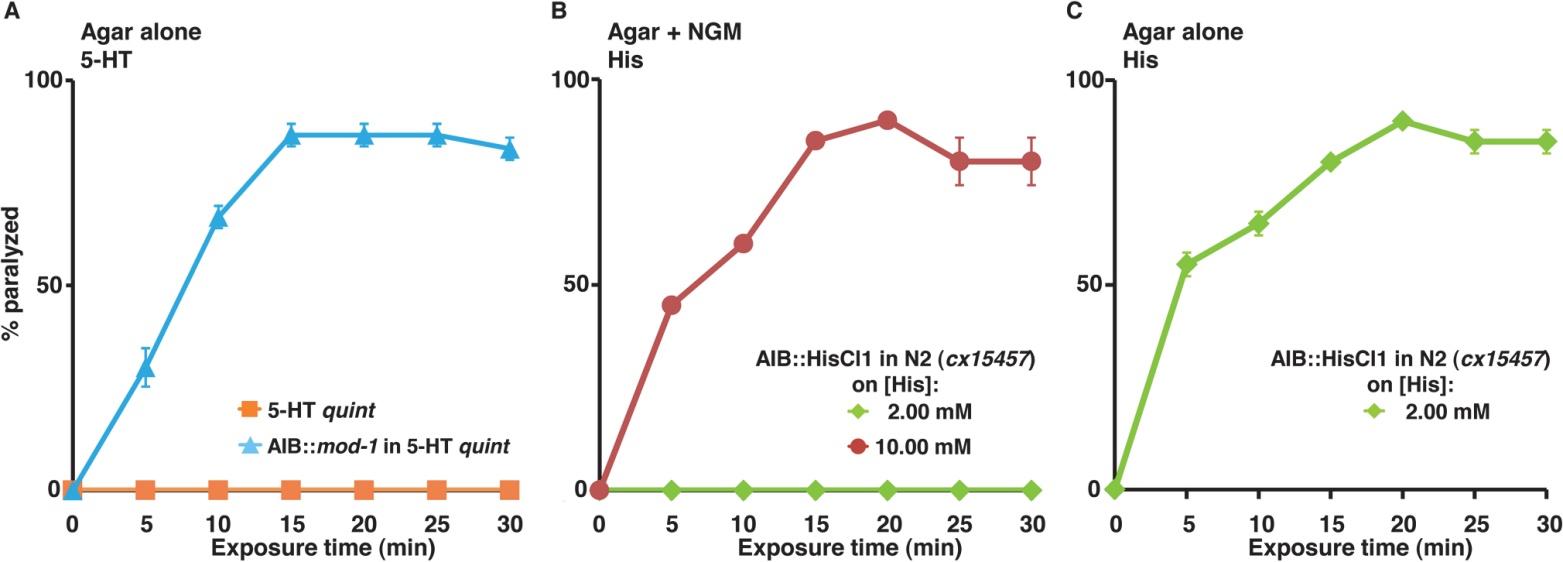
Inhibiting signaling from the two AIB interneurons causes “locomotory confusion” and paralysis. **A-C.** Paralysis of wild type, mutant and transgenic *C*. *elegans* on either NGM or non-NGM agar plates. **A.** 5-HT *quint* and 5-HT *quint* animals expressing MOD-1 in the AIBs (P*inx-1*) were examined for 5-HT (1 mM)-dependent paralysis on non-NGM agar plates, as outlined in Methods. Data are presented as mean ± SE (n = 3). **B and C.** Wild type animals expressing HisCl1 in the AIBs (*cx15457*) were examined for histamine (2 or 10 mM)-dependent paralysis on NGM (**B**) and non-NGM (**C**) agar plates. Data are presented as mean ± SE (n = 3).

Our results suggest that inhibiting AIB signaling by the expression of a Gα_o_-coupled 5-HT receptor in the AIBs of the 5-HT *quint* can cause paralysis (Fig 2B). Similarly, the AIB-specific expression (P*inx-1*) of the 5-HT-gated Cl^-^ channel, MOD-1 can also cause paralysis (Fig 6A). In contrast, ablation of the AIBs does not cause paralysis [56, 57]. Interestingly, the activation of a *Drosophila* histamine-gated Cl^-^ channel (HisCl1) expressed ectopically in the AIBs (*cx15457*) with 2 mM exogenous histamine (His) caused AIB hyperpolarization and locomotory phenotypes, but not paralysis [37]. In contrast, increasing the histamine concentration to 10 mM caused paralysis that persisted for up to 24 hrs in the presence of histamine [37]. Similarly, in the present study, 2 mM histamine did not cause paralysis in wild type animals or in transgenic animals expressing HisCl1 in the AIBs (*cx15457*) on NGM plates (Fig 6B). However, 2 mM histamine caused significance paralysis under the modified hypotonic assay conditions used in the present study or when the histamine concentration was raised to 10 mM on NGM plates (Fig 6B and 6C). Since the ablation of the AIBs does not cause paralysis, these results support our previous hypothesis that the partial inhibition of AIB signaling by partial hyperpolarization or the activation of Gα_o_ signaling causes an imbalance in the locomotory circuit that results in a state of decision-making “confusion,” an inability to execute and sustain unidirectional movement and ultimately, in cessation of locomotion (paralysis). Theoretically, any ligand that selectively unbalances AIB signaling has the potential to yield a similar locomotory phenotype and its target a potential site for anthelmintic development.

## Discussion

The monoamines, 5-HT, DA and TA each dramatically inhibit locomotion in *C*. *elegans* when applied exogenously at concentrations high enough to overcome the permeability barrier of the nematode cuticle, ultimately resulting in paralysis [24, 25, 27, 30]. In addition, monoamine-dependent locomotory paralysis is also observed in many parasitic nematodes, including *Ascaris suum* and *Heterodera glycines* [31, 32]. Using the *C*. *elegans* model, the receptors involved in monoamine-dependent locomotory inhibition have been identified and localized [22–30]. Interestingly, the key receptors involved in 5-HT, DA and TA inhibition each function at a different level in the locomotory circuit [24, 28, 30]. For example, 5-HT-dependent paralysis in *C*. *elegans* involves the expression of the Gα_o_-coupled, 5-HT_1_-like receptor, SER-4, and the 5-HT-gated Cl^-^ channel, MOD-1 in a limited number of interneurons, including the two AIBs [25]. Importantly, 5-HT_1_-like agonists appear to have anti-nematodal activity *in vivo* [34, 35]. Indeed, the results of the present study suggest that partial inhibition of the AIBs by activation of an endogenously expressed Gα_o_-coupled 5-HT_1_-like receptor or 5-HT-gated Cl^-^ channel, or a heterologously expressed histamine-gated Cl^-^ channel, interferes with AIB signaling, causes “locomotory confusion” and ultimately paralysis. Interestingly, animals with ablated AIBs are still motile and move efficiently, although their rates of spontaneous reversal are dramatically altered, suggesting either that this partial inhibition differentially affects AIBs signaling to cause locomotory paralysis or that the ablated animals have compensated for the loss of the AIBs [56, 57].

The present study provides further support to the use of “chimeric” *C*. *elegans*, created by the heterologous, ectopic expression of potential key drug targets from parasitic nematodes, for use as a platform for agonist identification and potential anthelmintic screening. Although the present is focused on inhibitory monoamine GPCRs and monoamine-gated ion channels, it can potentially be expanded to any signaling molecules for which the appropriate mutant backgrounds can be prepared. Specific promoters are available for *C*. *elegans* muscles and most neurons; alternatively, specific promoters to other neurons can be generated using a Cre-Lox approach [58]. This screening system has the potential to combine the individual pharmacologies of the receptors from different parasitic nematodes with the environment and accessory proteins necessary for functional expression. This becomes especially important because nematode-specific cells lines are not available and the expression of nematode receptors in mammalian cells is quite variable and can require a host of additional modifications, including temperature shock to achieve expression [59, 60]. In fact, few studies have compared receptor pharmacologies *in vivo* with those of the nematode receptors heterologously expressed in mammalian cells. The functional reconstitution of nematode receptors in heterologous systems (*Xenopus* oocytes, *etc*.) often requires additional accessory proteins and/ or subunits that might not have been identified previously, hindering the further development of potential drug targets [61–64]. Not only do transgenic *C*. *elegans* provide a promiscuous expression platform for distantly-related receptors: these ectopically-expressed receptors are functional and appear to maintain their ligand-receptor specificity, as highlighted above where only the transgenic animals expressing the human receptor were paralyzed by 8-OH-DPAT. The identification of DOP-3 as a secondary target in PAPP-dependent paralysis also validates the utility and convenience of transgenic *C*. *elegans* as a platform for drug target identification and potential anthelmintic screening. Although the current study uses transgenic animals expressing the desired receptor as an extra-chromosomal array, stable lines can be readily constructed if required [65].

This screening platform also includes the nematode cuticle, a potential barrier to the entry of any anthelmintic, as well as a wide array of ABC transporters involved in drug efflux and resistance [66]. The cuticle is made up of six layers, the epicuticle, external cortical, internal cortical, medial, fiber and basal, as well as a carbohydrate-rich surface coat external to the epicuticle [67]. The lipid-rich epicuticle layer might be the key barrier to externally-applied drugs, especially water-soluble molecules (5-HT, TA, 8OH-DPAT *etc*.) and the reason for the high concentration required to cause paralysis under isotonic environment, *i*.*e*. on NGM agar plates [52, 67, 68]. As mentioned, although *C*. *elegans* cuticle appears to be more impermeable than those of some parasitic nematodes, the permeability of the *C*. *elegans* cuticle can be manipulated by modifying incubation conditions and the availability of various mutant backgrounds. By incubating the animals in a salt-free, hypotonic environment, 5-HT paralyzes wild-type animals with an EC_50_ of about 0.5 mM, in contrast with an EC_50_ of about 12 mM on isotonic NGM agar plates. In addition, a number of *C*. *elegans* mutations that appear to have increase cuticular permeability may also be useful for enhancing small molecule screening against an array of medically-important targets, including those involved in locomotory paralysis [54, 69]. For example, many of the *bus* (bacterially swollen) mutations appear to alter the cuticle and increase permeability [54]. Indeed, as shown in Fig 1E and 1F, it might be possible to select specific cuticle mutants with permeabilities that mimic those of individual parasitic nematodes, providing a mean to bypass complicated and expensive process of culturing live parasites, at least during preliminary stages of agonist screening. In fact, *C*. *elegans* has been used in the past for large-scale small molecule screens and chemical genomics and predictive models for drug accumulation and bioactivity have been developed that may be used to bias preliminary screening [70, 71]. This ability to alter cuticular permeability will certainly be useful for agonist and potential anthelmintic identification, but in the case of the monoamines examined, relatively high concentrations of ligand are still required and, ultimately, any potential agonists identified using this approach will have to be validated in the target of choice.

In summary, this study has identified two key AIB interneurons that play a role in 5-HT-dependent paralysis and suggests that partial inhibition of signaling from the neurons has the potential to cause “locomotory confusion,” and paralysis. In addition, these studies have demonstrated and validated the utility of these “chimeric” *C*. *elegans* as a platform for agonist identification and potential anthelmintic screening.

## Supporting Information

S1 Text Primers for making fusion constructs. (DOCX)
