# Supplementary material for: Heterologous Expression in Remodeled C. elegans: A Platform for Monoaminergic Agonist Identification and Anthelmintic Screening
Source: PLoS Pathog. 2015 Apr 30;11(4):e1004794. doi: 10.1371/journal.ppat.1004794 (PMC4415803; doi:10.1371/journal.ppat.1004794)
Supplement: S1 Text — (DOCX) [file ppat.1004794.s001.docx]

**S1: Primers for making fusion constructs**

**P*npr-9*::*ser-4*::GFP**

-R npr-9::ser-4

5’-GAGAAGCGTCTCGTCGATCATTTCCCAGGAAGTAGCTCTAAAATTACAATAAAGACG-3’

-F npr-9p::ser-4

5’-GTAATTTTAGAGCTACTTCCTGGGAAATGATCGACGAGACGCTTCTCAATCTCACGG-3’

-R ser-4::GFP

5’-CAGTGAAAAGTTCTTCTCCTTTACTCATATAATCGTGAATAAGGCACATCCGTTTGATG-3’

-F ser-4::GFP

5’-CAAACGGATGTGCCTTATTCACGATTATATGAGTAAAGGAGAAGAACTTTTCACTGG-3’

**P*unc-17β*::*ser-4*::GFP**

-R unc-17β::ser-4

5’-GAGAAGCGTCTCGTCGATCATAAGGGTCCTCCTGAAAATG-3’

-F unc-17β::ser-4

5’-CATTTTCAGGAGGACCCTTATGATCGACGAGACGCTTCTCAATCTCACGG-3’

**P*unc-17β*::*Drosophila* 5-HT_1_-like (5-HT1A)::GFP**

-R unc-17β::Dro 5-HT1A

5’-GCTGGTCTCGTGCGCCATAAGGGTCCTCCTGAAAATGTTCTATGTTATGTTAG-3’

-F unc-17β::Dro 5-HT1A

5’-GAACATTTTCAGGAGGACCCTTATGGCGCACGAGACCAGCTTTAATG-3’

-R Dro 5-HT1A::GFP

5’-GAAAAGTTCTTCTCCTTTACTCATGAGCTTCCCGCTGCGGTAGTGG-3’

-F Dro 5-HT1A::GFP

5’-CTACCGCAGCGGGAAGCTCATGAGTAAAGGAGAAGAACTTTTCACTGG-3’

**P*unc-17β*::Human 5-HT_1A_ (HTR1A)::GFP**

-R unc-17β::Hum HTR1A

5’-CAGGGCTGAGCACATCCATAAGGGTCCTCCTGAAAATGTTCTATG-3’

-F unc-17β::Hum HTR1A

5’-GAACATTTTCAGGAGGACCCTTATGGATGTGCTCAGCCCTGGTC-3’

-R Hum HTR1A::GFP

5’-CAGTGAAAAGTTCTTCTCCTTTACTCATCTGGCGGCAGAACTTACACTTAATG-3’

-F Hum HTR1A::GFP

5’-GTGTAAGTTCTGCCGCCAGATGAGTAAAGGAGAAGAACTTTTCACTGG-3’

**P*unc-17β*::*mod-1*::GFP**

-R unc-17β::mod-1

5’-GTAGTGTGATTTCAGGAATAAACTTCATAAGGGTCCTCCTGAAAATGTTCTATG-3’

-F unc-17β::mod-1

5’-GAACATTTTCAGGAGGACCCTTATGAAGTTTATTCCTGAAATCACACTACTCTTGC-3’

-R mod-1::GFP

5’-GTTCTTCTCCTTTACTCATCTGATAGTTTTGATCGAAAGTTTGAGAC-3’

-F mod-1::GFP

5’-CTTTCGATCAAAACTATCAGATGAGTAAAGGAGAAGAACTTTTCACTGG-3’

**P*unc-17β*::*H. contortus* *mod-1*::GFP**

-R unc-17β::Hco mod-1

5’-GGTGAATAGCAATTCACGTATCATCATAAGGGTCCTCCTGAAAATGTTCTATG-3’

-F unc-17β::Hco mod-1

5’-GAACATTTTCAGGAGGACCCTTATGATGATACGTGAATTGCTATTCACCTTGC-3’

-R Hco mod-1::GFP

5’-GAAAAGTTCTTCTCCTTTACTCATGACGATATAATTCCTCACATTAAAAAATGTAAACGC-3’

-F Hco mod-1::GFP

5’-CATTTTTTAATGTGAGGAATTATATCGTCATGAGTAAAGGAGAAGAACTTTTCACTGG-3’

**P*myo-3*::*H. contortus* *mod-1*::GFP**

-R myo-3::Hco mod-1

5’-GGTGAATAGCAATTCACGTATCATCATTTCTAGATGGATCTAGTGGTCGTGGG-3’

-F myo-3::Hco mod-1

5’-CACGACCACTAGATCCATCTAGAAATGATGATACGTGAATTGCTATTCACCTTGC-3’

**P*myo-3*::*lgc-55*::GFP**

-R myo-3::lgc-55

5’-GTAAGAATGAACGAGAACACCATTTCTAGATGGATCTAGTGGTCGTG-3’

-F myo-3::lgc-55

5’-CGACCACTAGATCCATCTAGAAATGGTGTTCTCGTTCATTCTTACTTTTACC-3’

-R lgc-55::GFP

5’-GTTCTTCTCCTTTACTCATATCCTTGGATTTTGCCGTATAATACATCC-3’

-F lgc-55::GFP

5’-GTATTATACGGCAAAATCCAAGGATATGAGTAAAGGAGAAGAACTTTTCACTGG-3’

**P*unc-17β*::*H. contortus* *lgc-55*::GFP**

-R unc-17β::Hco lgc-55

5’-GGTGAGAATGAAGGTGAAAGGCATAAGGGTCCTCCTGAAAATGTTCTATG-3’

-F unc-17β::Hco lgc-55

5’-GAACATTTTCAGGAGGACCCTTATGCCTTTCACCTTCATTCTCACCTTTACC-3’

-R Hco lgc-55::GFP

5’-GTGAAAAGTTCTTCTCCTTTACTCATATCTTTTGACTTTGCTGTATAGTACATCCAATAG-3’

-F Hco lgc-55::GFP

5’-GGATGTACTATACAGCAAAGTCAAAAGATATGAGTAAAGGAGAAGAACTTTTCACTGG-3’

**P*inx-1*::*mod-1*::GFP**

-R inx-1::mod-1

5’-GATTTCAGGAATAAACTTCATTCCGGCGGACAAGAACTGCAATGAAAAC-3’

-F inx-1::mod-1

5’-CATTGCAGTTCTTGTCCGCCGGAATGAAGTTTATTCCTGAAATCACACTACTC-3’

**P*dop-3*::*dop-3* RNAi**

-Rs Pdop-3::dop-3 Rs

5’-CGTGGTGTTGTCCAGCCAACATTCTGATTTTAGACGACGGATTTCCAGAG-3’

-Ras Pdop-3::dop-3 Ras

5’-CGAACGGATCAAATACTGTTAGAAATCTCTGATTTTAGACGACGGATTTCCAGAG-3’

-TF dop-3 F

5’-ATGTTGGCTGGACAACACCACGTTAC-3’

-TFi dop-3 Fi

5’-GCTGGACAACACCACGTTACAGAC-3’

-TR dop-3 R

5’-GATTTCTAACAGTATTTGATCCGTTCGATTTTTG-3’

-TRi dop-3 Ri

5’-CAGTATTTGATCCGTTCGATTTTTGAAATGTTTGC-3’
